# Recurrence of pericarditis after influenza vaccination: a case report and review of the literature

**DOI:** 10.1186/s40360-018-0211-8

**Published:** 2018-05-05

**Authors:** Riccardo Mei, Emanuel Raschi, Elisabetta Poluzzi, Igor Diemberger, Fabrizio De Ponti

**Affiliations:** 1 Department of Medical and Surgical Sciences, Alma Mater Studiorum-University of Bologna, 40126 Bologna, Italy; 2 Department of Experimental, Diagnostic and Specialty Medicine, Alma Mater Studiorum-University of Bologna, 40126 Bologna, Italy

**Keywords:** Pericarditits, Rechallenge, Adverse event following immunization, Influenza vaccine, Case report

## Abstract

**Background:**

This case report describes a patient with pericarditis likely attributed to influenza vaccination (positive rechallenge), with a literature review.

**Case presentation:**

A 87-year old patient developed pericarditis after influenza vaccination, with acute chest pain, without ECG abnormalities or increased cardiac enzyme levels. Echocardiogram showed moderate pericardial effusion. Recovery was obtained through steroids One year later, few days after re-immunization, the patient experienced the same symptoms and was admitted to hospital with diagnosis of recurrence of pericarditis with severe pericardial effusion, again treated with steroids. Other possible causes were ruled out and the cardiologist recommended against influenza vaccinations in the future; the patient did not experience recurrence of pericarditis in the following 6 years. Cases of pericarditis following influenza immunization in the literature were also reviewed.

**Conclusions:**

Pericarditis following immunization for influenza is very rarely reported in the literature. In a few cases, influenza vaccination seems likely responsible. We suggest considering recent immunization in patient’s history as part of the differential diagnosis in elderly with chest pain.

## Background

Cardiac complications after immunization are extremely rare events, albeit clinically important. In the past, the association between an increased risk of myocarditis and the use of smallpox vaccine, both in military personnel and civilians, drew the attention of clinicians and regulators [1]. Cases of myocarditis, pericarditis or myopericarditis after other vaccines, including multiple immunization have been reported as well, but a definite association was never demonstrated [2–4]. Here below, we describe a case of established pericarditis in an elderly patient following seasonal influenza vaccine with unintentional re-challenge. A 6-year follow-up free of vaccine is also reported. A MEDLINE review was also performed in February 2017 to discuss published case reports/series.

## Case presentation

**Fig. 1:**
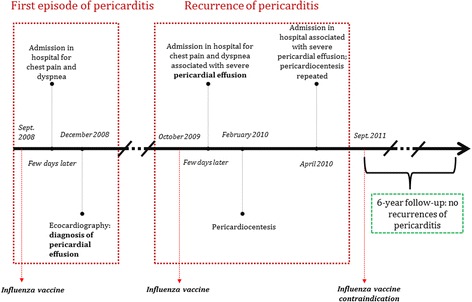
Timeline of events

On September 2008 a 87-year-old man was admitted to hospital with diffuse, worsening (especially at night) chest pain for several days (Fig. 1). His past history included thyroidectomy, acute myocardial infarction and total atrioventricular block with sick sinus syndrome for which a single-chamber pacemaker was implanted and subsequently upgraded to dual-chamber pacemaker (because of pacemaker syndrome). He was also affected by chronic obstructive pulmonary disease (no treatment reported) and mild chronic kidney failure. Five months before (April 2008) he had been admitted to hospital for worsening heart failure due to acute pneumonia; atrial fibrillation also had occurred and warfarin was started.

In the emergency department, physical examination revealed diminished heart sounds, while chest X-rays showed cardiomegaly. No ECG abnormalities or increased cardiac enzyme levels were found. The primary diagnosis was heart failure due to coronary insufficiency and nitroglycerin provided rapid relief.

**Fig. 2:**
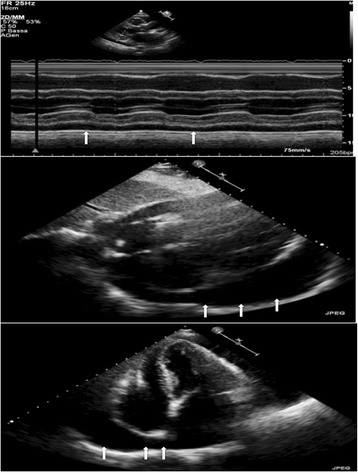
Transthoracic echocardiogram (M-Mode, subcostal four-chamber and apical four-chamber view) showing moderate pericardial effusion (arrows)

Three months later (December 2008), in a follow-up visit echocardiogram showed moderate pericardial effusion without signs of tamponade (Fig. 2). Steroid therapy (prednisone 40 mg/day with tapering for 1 month) was initiated because of colchicine-related gastrointestinal toxicity (discontinued early after first administration). *T. pallidum* immunoglobulin G (IgG)/IgM antibody, hepatitis-C virus IgG antibody, hepatitis-B virus surface antigens and human immunodeficiency virus p24-antigen and antibodies were negative.

One year later (October 2009), the patient’s conditions worsened due to onset of chest pain and dyspnea and, on hospital admission, echocardiography confirmed pericarditis with severe pericardial effusion; steroid therapy (as above) was resumed and after 4 months pericardiocentesis was performed.

In April 2010 the patient was again admitted to hospital for pericarditis relapse and knee-arthritis with important synovial effusion. Periocardiocentesis was repeated and 750 cm^3^ of serosanguineous fluid was drained; arthrocentesis was also performed. Fluid cultures were both negative for aerobic and anaerobic bacteria. Mycobacteria culture and Quantiferon test were negative for tuberculosis infection as well as cytologic test for malignant cells.

In order to find out the possible cause of pericarditis, a careful history ascertained that the patient had received influenza vaccine (Influvac®,trivalent, inactivated) 5–7 days before the first admission; the patient reported re-immunization with influenza vaccine (unknown name and typology) also one year later, just some days before the second admission to hospital. In the absence of other possible causes (infectious diseases, autoimmune diseases, cancer, drugs), hypothesis of pericarditis and recurrence caused by influenza vaccine was therefore considered. Following the last hospital admission the cardiologist in charge recommended against influenza vaccinations in the future and the patient did not experience recurrence of pericarditis in the following 6 years of follow-up.

## Discussion and conclusions

Annual vaccination is an important public health measure to prevent influenza because influenza viruses change their antigenic characteristics frequently and may easily spread in the population [5–8]. In particular, elderly population (≥65 years old) and individuals with underlying diseases have either higher risk of complications of influenza and death. In these categories, influenza vaccine reduces dramatically both influenza occurrence and the severity of the illness [9, 10]. Moreover, influenza vaccine in older people is associated with a lower risk of cardiovascular events, especially in those with underlying cardiovascular risk factors (e.g. coronary disease) [11]. Annual immunization against influenza also reduces risk of death particularly in previously immunized [12] subjects.

Pericarditis typically presents with chest pain (> 85–90% of cases), pericardial friction rub (≤33% of cases), ECG changes (up to 60% of cases), increased cardiac enzymes and markers of inflammation (i.e. C-reactive protein, erythrocyte sedimentation rate and white blood cell count). Recurrent pericarditis is diagnosed when a symptom-free interval of 4–6 weeks or longer separates the first episode of pericarditis. Recurrence occurs in 15–30% of cases after an initial episode, and may increase to 50% after a first recurrence in patients not treated with colchicine, particularly if treated with corticosteroids [13].

We searched MEDLINE in February 2017 for case reports/series, by combining the following key-words: “influenza”, “vaccine”, “immunization” and “pericarditis”, without language restrictions. Six case reports (7 patients) of pericarditis after influenza immunization were identified: of these, 4 patients aged more than 60 years, mean time of symptom onset was 7 days, outcome was favorable in all the cases (recovery). According to the authors, a positive association between pericarditis and vaccine was defined in 4 cases, while 3 cases resulted with a “possible” association. In only one case, recurrence of pericarditis after influenza immunization reported, about 2 years after the first episode. Further details are shown in Table 1. Only one case described a patient with recurrent pericarditis after influenza vaccination in two consecutive years [14]. Interestingly, Zanettini et al. compared 84 patients with pericarditis, divided into two groups; patients immunized with influenza vaccine, compared with patients not immunized, had a typical seasonal distribution with a peak in autumn, fewer comorbidities, less exuberant signs and symptoms, and clinical relief with NSAIDs use [15]. Considering the widespread use of influenza vaccination (approximately two of every five children and adults in the United States were vaccinated by early November 2016) [16], the occurrence of iatrogenic pericarditis is an extremely rare complication. However, in the light of the described event and the series reported by Zanettini et al., we cannot exclude that similar cases may be underestimated since the cause of more than a half pericarditis (86.0% reported by Permanyer-Miralda et al., 78.0% reported by Zayas et al. and 83.2% reported by Imazio et al.) [17–19] remains undetermined.

**Table 1:** MEDLINE search results for pericarditis after influenza vaccination

Year	Age and sex	Adverse reaction	Mean time to onset	Association according to the author	Evolution	Ref.
1981	61 F	Recurrent pericarditis	7 days	Yes	Favorable	[14]
1997	40 M	Pericarditis	5 days	Yes	Favorable	[22]
2000	75 M;40 M	Pericarditis	6 days; 5 days	Yes	Favorable (both)	[23]
2003	87 M	Pericarditis	5 days	Possible	Favorable	[24]
2004	68 F	Pericarditis and Guillain-Barré syndrome	2 weeks	Possible	Favorable	[25]
2004	≈60 (23 cases; 16F and 7 M)	Pericarditis	Not reported	Yes	Favorable	[15]
2008	59F	Pericarditis, pancytopenia, autoimmune haemolytic anemia, hepatitis and nephritis due to systemic systemic lupus erythematosus flare-up	1 month	Possible	Favorable	[26]

We described a rare case of pericarditis following immunization, which did not meet the typical clinical and laboratory presentation. The underlying chronic diseases of the elderly patient (ischemic cardiomyopathy, permanent atrial fibrillation, chronic obstructive pulmonary disease and mild chronic kidney failure) do not fully explain pericarditis, although they probably contributed to its appearance (e.g. use of anticoagulant therapy). We cannot exclude that a recurrent viral infection could have caused the pericarditis episodes, because no specific viral studies were performed to systematically rule out viral etiologies of pericarditis. Nonetheless, hepatitis and HIV infections were excluded, as well as bacterial infections, since laboratory data and pericardial fluid culture were negative. These findings, together with positive re-challenge (re-administration one year after the first event with event re-occurrence) are key elements to define the association with influenza vaccine as “correlated” by WHO-AEFI (Adverse event Following Immunization) algorithm [20]. Moreover, the patient was monitored for the following 6 years during which influenza vaccination was not performed and no recurrence of pericarditis was reported (positive de-challenge). This is in line with Engler et al. [21] who strengthened the importance of active long-term surveillance.

In conclusion, although under-reporting can be supposed, the rarity of pericarditis after immunization does not affect the already established positive benefit-risk profile of influenza vaccine (and of vaccines in general). However, considering the high prevalence of pericarditis of undetermined origin, episodes of recent immunization should be highlighted in patient’s history to support differential diagnosis.

## Abbreviations

AEFI: Adverse event Following Immunization
ECG: Electrocardiogram
NSAIDs: Nonsteroidal anti-inflammatory drugs
WHO: World Health Organization
